# Update on *GNA* Alterations in Cancer: Implications for Uveal Melanoma Treatment

**DOI:** 10.3390/cancers12061524

**Published:** 2020-06-10

**Authors:** Lionel Larribère, Jochen Utikal

**Affiliations:** 1 Skin Cancer Unit, German Cancer Research Center (DKFZ), 69120 Heidelberg, Germany; j.utikal@dkfz.de; 2 Department of Dermatology, Venereology and Allergology, University Medical Center Mannheim, Ruprecht-Karl University of Heidelberg, 68167 Mannheim, Germany

**Keywords:** uveal melanoma, GNA mutations, G proteins, GPCR, therapy

## Abstract

Tumorigenesis is correlated with abnormal expression and activity of G protein-coupled receptors (GPCRs) and associated G proteins. Oncogenic mutations in both GPCRs and G proteins (*GNAS*, *GNAQ* or *GNA11*) encoding genes have been identified in a significant number of tumors. Interestingly, uveal melanoma driver mutations in *GNAQ*/*GNA11* were identified for a decade, but their discovery did not lead to mutation-specific drug development, unlike it the case for *BRAF* mutations in cutaneous melanoma which saw enormous success. Moreover, new immunotherapies strategies such as immune checkpoint inhibitors have given underwhelming results. In this review, we summarize the current knowledge on cancer-associated alterations of GPCRs and G proteins and we focus on the case of uveal melanoma. Finally, we discuss the possibilities that this signaling might represent in regard to novel drug development for cancer prevention and treatment.

## 1. Introduction

The G protein-coupled receptor (GPCR) family of proteins comprises more than 800 members and their seven-transmembrane domain structure allows, after binding to their ligands, the activation of heterotrimeric G proteins, which generates second messengers, as well as kinase cascades activation in the cytoplasm of the cells. These signals ultimately control gene transcription, cell survival, motility, and growth. It is nowadays clearly established that signals transmitted by GPCR/G proteins and downstream targets are involved in the initiation and progression of cancer. For example, overactivation of pathways such as AKT/mTOR, MAPK and Hippo will lead to altered cell growth and survival. In addition, invasion of cancer cells will be favored by GPCR-regulated RHO GTPases activation and changes in the cytoskeleton. GPCR/G proteins have also been described to play a role in angiogenesis and organization of tumor microenvironment.

Therefore, our knowledge of how GPCR/G proteins are responsible for the development of cancer transformation is crucial to identify new therapeutic targets. In this review, we describe first the G protein-encoding gene alterations that have been identified in malignancies. Second, we focus on the role of alterations in *GNAQ/11* specifically in uveal melanoma. We finally discuss the opportunities offered by altered GPCR/G protein signaling in cancer to develop rational treatment strategies for patients with advanced uveal melanoma.

## 2. Heterotrimeric G Proteins 

G proteins are guanine-nucleotide-binding proteins, which play a key role in signal transduction. When bound to GTP, G proteins are active, however an intrinsic GTPase activity allows their inactivation in the GDP-bound status. These heterotrimeric G proteins consist of α-, β- and γ-subunits. The activation of G proteins involves several mechanisms including the stimulation of seven-transmembrane domain receptors (GPCRs), of tyrosine kinases receptors (TKRs) or the activation of guanine-nucleotide exchange factors (GEFs) [1]. 

**Figure 1:**
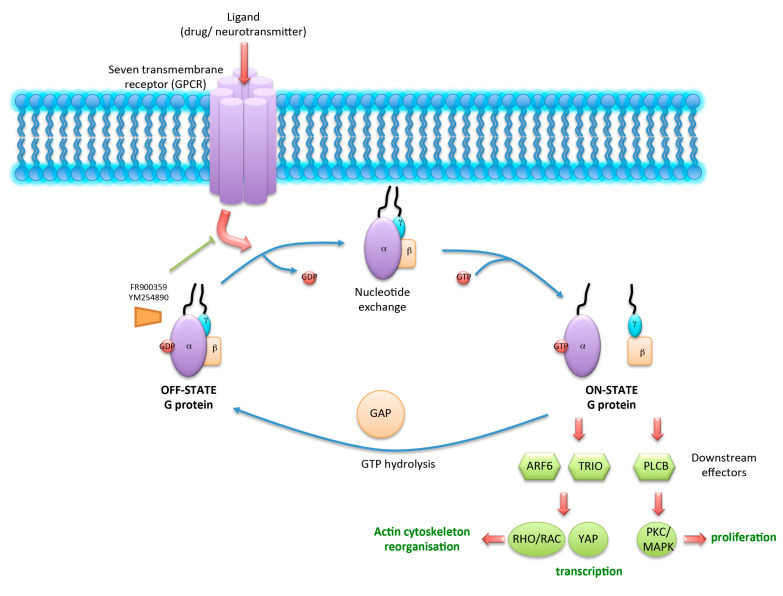
Schematic of G protein activation after G protein-coupled receptors (GPCR) binding to its ligand. Ligand-activated GPCR allows the release of GDP from OFF-STATE G proteins. “Empty pocket“ will be refilled promptly with GTP. This results in the disassembly of Gα from Gβγ subunits (ON-STATE) and activation of downstream effectors such as ARF6, TRIO, and PLCβ. Cellular pathways such as RHO/RAC or YAP are involved in actin cytoskeleton reorganization and cell growth. PKC/MAPK controls cell proliferation. GTPase function leads to GTP hydrolysis and reformation of the inactive heterotrimer. This step is regulated by GTPase-activating proteins (GAPs). FR and YM inhibitors block G protein signaling by preventing GDP release.

In the case of GPCRs stimulation and after conformation change of the receptors, the Gα unit will load GTP instead of GDP, leading to its release from the Gβγ unit and from the receptor. GTP-bound Gα and Gβγ will subsequently activate their cognate effectors. Activated G proteins will transmit the signal from several hormones and control many cell functions, including transcription, motility and secretion. This process is tightly regulated temporally and spatially, and leads to the activation of a panel a multiple G protein-specific targets (Figure 1). For example, ARF6 (ADP-ribosylation factor 6), TRIO, and PLCβ (phospholipase C β) represent downstream effectors which activate cellular pathways such as RHO/RAC (RAS-related C3 botulinum toxin) or YAP (yes-associated protein), which are involved in actin cytoskeleton reorganization. PKC (protein kinase C)/MAPK can be activated by PLCβ and controls cell proliferation [2,3]. Therefore, GPCRs are considered as molecular rheostats and represent potential targets for therapeutic inhibition [4]. 

## 3. Mutations in Genes Encoding G Proteins

The *GNA* gene family contains several members, which encode for G proteins including *GNAS* (a complex locus that encodes Gαs subunits), *GNA11* (encoding Gα11 subunits), and *GNAQ* (encoding Gαq subunits). Oncogenic mutations in these genes usually impair their GTPase activity, leading to constitutively active forms of GTP-bound proteins and to extended downstream signaling. For example, in the context of McCune–Albright syndrome, an active form of Gαs promotes cellular hyperproliferation [5]. Nonetheless, it was recently suggested that Gαs gain-of-function mutation can bypass the need for GTP binding and directly activate GDP-bound Gαs [6].

### 3.1. GNAS Mutants

Based on deep sequencing studies, it is known that mutations in *GNAS* occur in a wide range of tumors. Table 1 represents the frequency of alterations in this gene which were found in the The Cancer Genome Atlas TCGA PanCan 2018 [7,8] (Table 1). Most frequently mutated entities (approximately 10%) were colorectal, stomach and uterine cancers. Least mutated (<1%) were glioma, lymphoma and germinal cell cancers. With a 4% rate, *GNAS* is the most frequently altered G protein-encoding gene in human tumors. Most of these mutations were identified on two particular hotspot regions—R201 and Q227 [9,10,11]. 

Interestingly, *GNAS* is described as a driver oncogene in a subset of colon adenomas and adenocarcinomas [12], and mutations in this gene can promote hyperplasia of endocrine cells in thyroid and pituitary tumors [9]. In addition, Gαs was shown to regulate inflammatory mediators such as cyclooxygenase 2 (COX2)-derived prostaglandins [13]. Since *COX2* has a known protumorigenic role in colon neoplasia, oncogenic mutations in *GNAS* may therefore activate a proinflammatory response, which favors tumor development [14,15]. 

### 3.2. GNAQ and GNA11 Mutants

*GNAQ* and *GNA11* genes are two paralogs and share 90% sequence homology. *GNAQ* and *GNA11* mutations are mutually exclusives and occur in about 2% of human cancers. Mutations in *GNAQ* and *GNA11* were mostly identified at residues involved in GTPase activity (Q209 or R183) [16,17]. Apart from being found in the meninges (59%), in blue naevi (83%), and in a subset of cutaneous melanomas linked to chronic sun-induced damage (3–4%), *GNAQ* or *GNA11* represent driver oncogenes in around 50% uveal melanoma [17,18,19]. Details on the role of these mutations in uveal melanoma will be discussed in the next paragraph. 

### 3.3. Other GNA Mutants

Several mutations in other Gα encoding genes have been identified at a much lower frequency in human cancers and might not be activating. Therefore, their exact consequence is not yet known. As an example, *GNA15* (encoding Gαq subunits) is significantly mutated in skin melanomas that do not often carry *GNAQ* or *GNA11* mutations [20].

Interestingly, very few mutations on Gβ and Gγ subunits have been identified compared to Gα subunits. However, mutations in GPCRs encoding genes were identified in almost 20% human cancers. Although many of these mutations are still uncharacterized regarding the impact on tumorigenesis, the most frequent were found in the thyroid-stimulating hormone receptor (TSHR), smoothened (SMO), glutamate metabotropic receptors (GRMs), the lysophosphatidic acid receptor (LPA) and the sphingosine-1-phosphate (S1P) receptor. More details on mutations in GPCRs encoding genes are described in the review of Kan et al. [21]. At the molecular level, the Hippo-YAP pathway was described to be essential to tumorigenesis downstream the activation of GPCRs [2,22]. A CRISPR/Cas9-based systematic analysis showed that cell sensitivity to the morphogen Sonic Hedgehog (SHH) can be regulated by GPCR-induced signals (i.e., SMO), leading to GNAS and PKA activation [23].

In sum, hotspot mutations in *GNAS*, *GNAQ* and *GNA11* have been identified in human tumors; however, a better characterization of the mutations on the other G protein-encoding genes is still needed. Nevertheless, common signaling between G proteins and their activating GPCRs could represent therapeutic opportunities for cancer treatment.

## 4. Recently Described Roles of G Protein Mutations in Tumorigenesis

Cellular migration and motility was recently described as a new role of mutations of G proteins. Cervantes-Villagrana and colleagues demonstrated that Gαq and Gα13 proteins can directly regulate the signaling of Gβγ to PREX1 [25]. Since RAC and RHO GTPases exert opposing effects, the authors dissected their respective activating signalings via Gα12/13 or Gαq. By means of pharmacological inhibition, they showed that Gαq activates PREX1 more efficiently under the activation of lysophosphatidic acid receptors. Moreover, by using different Gα variants in which GTPase activity was lost, they could observe the formation of stable complexes with Gβγ and a prevention of its downstream interaction with PREX1. In sum, the authors identified a new mechanism of the prioritization of RHO over RAC mediated by Gαq and Gα13.

Another study showed that Giα2 plays a role in the cell migration downstream of PI3K/AKT and RAC1 [26]. Silencing of Giα2 in pancreatic cancer cells reduced the migration dependent on TGFβ, oxytocin, SDF-1α, and EGF. In addition, silencing of Giα2 in cells that overexpressed active RAC1 abolished their migration without affecting the basal RAC1 activation.

Metabolism of pancreas and in particular lipid reprogramming has recently been described to play a key role in *GNAS*-driven pancreatic tumorigenesis [27]. Indeed, genetically engineered mouse models could show cooperation between *Gnas^R201C^* and *Kras^G12D^* mutations to promote the initiation of intraductal papillary mucinous neoplasms (IPMNs), the latter progressing into pancreatic ductal adenocarcinomas (PDAs) after additional *Tp53* loss. The authors not only observed an essential role of mutant *Gnas* in tumor maintenance but also a mechanism of protein kinase A-mediated suppression of salt-inducible kinases (*Sik1*-*3*), in association with lipid remodeling.

As increased intracellular ROS levels are known to induce cell cycle arrest, senescence, and apoptosis, the deregulation of ROS levels may lead to tumorigenesis. Hydrogen peroxide, and superoxide are the main products of NADPH oxidase (NOX enzymes) and are considered as signaling molecules [28]. Interestingly, angiotensin II receptor 1 (AT1R) has been shown to activate NOX1 likely via Gαq and PLCβ which activate PKC [29]. Evidence showed that thrombin can increase NOX1-dependent ROS generation by mechanisms involving PAR4 or EGFR transactivation [24,30,31]. Several studies have reported an upregulation of NOX4 in response to GPCR ligands such as angiotensin II (AT-II), urotensin-II, β-adrenergic agonists, renin and thrombin [32,33,34,35,36,37]. Interestingly, a direct link between GPCR and NOX4 transcription was described and involved transcription factors HIF1 and FoxO3a [35,38]. Similar to NOX1, NOX4-dependent ROS generation activated by AT-II involves Gαq/11, PLC activation, an increase in intracellular calcium levels and activation of PKC [39]. GPCR-induced cytosolic calcium levels are expected to regulate NOX5-dependent ROS generation. Thrombin, AT-II and endothelin 1 were reported to activate NOX5 by a mechanism which involved calmodulin recruitment and CAMKII activation [40,41,42,43]. NOX5 was also shown to be induced by bile acid activation of the TGR5 receptor and Gα [44,45]. GPCR can also generate ROS via small G-proteins (RAC) and activate JAK /STAT-dependent transcription [46]. 

Nevertheless, the precise identification of which G-protein is involved in GPCR-mediated ROS production is still unclear. A better understanding is expected in the near future based on the current engagement in G-protein targeted drug discovery.

Epigenetic mechanisms such as *GNA* gene promoter hypermethylation was proposed as a promising biomarker for hepatocellular carcinoma (HCC) diagnosis and targeted therapy [47]. Downregulation of *GNAO1* is associated with the early process of HCC and is a consequence of its promoter’s methylation, as observed by 5-Aza-2’-deoxycytidine (DAC) and trichostatin A (TSA) treatment. Moreover, this mechanism seems to be regulated by methyltransferase 1 (DNMT1).

Interestingly, a new study based on clinical data and next-generation sequencing (NGS) on a cohort of 1348 patients with a wide range of cancers presented the most frequent coalterations in the presence of *GNA* alterations (*GNAS*, *GNAQ*, and *GNA11*) to be in *AURKA*, *SRC*, *CBL* and *LYN* genes [48]. In sum, multiple recent studies indicate a key role of *GNA* mutations in processes such as cell migration, promoter hypermethylation and ultimately tumorigenesis.

## 5. G protein Mutations in Uveal Melanoma

The Cancer Genome Atlas (TCGA) has classified cutaneous melanoma tumors based on the most frequent genetic subtypes. Four main groups have been proposed as follows: mutant *BRAF*, mutant *RAS*, mutant *NF1*, and triple *BRAF*/*RAS/NF1* wildtype (which includes mutations in other genes such as *GNA*). 

Several recent studies bring evidence supporting a key role of *GNA* mutations specifically in the transformation of the melanocyte lineage. For example, several zebrafish models of uveal melanoma have shown that melanocyte-specific expression of driver mutations *GNAQ/GNA11(Q209L)* led to considerable changes in the melanocyte biology of the fish [49]. Moreover, the additional loss of tumor suppressor *Tp53* induced the development of melanocytic tumors including uveal melanoma, with almost complete penetrance. As observed in human uveal melanoma, the authors could find a nuclear localization of YAP, which could lead to transcription of genes involved in tumor growth [2,3]. Finally, they observed hyperpigmentation and altered melanocyte migration and survival, independently of *Tp53*. In a mouse model of leptomeningeal melanocytic neoplasms, the inducible expression of *Gnaq(Q209L)* variant at the neural crest stage before melanocyte differentiation, could favor the development of blue nevus-like lesions in the dermis, various melanocytic neoplasms in the cranium and spine but also melanoma of the central nervous system [50]. Interestingly, the authors observed different phenotypes depending on the time window of mutant expression or depending on several melanocyte precursor-specific promoters used to express it. Therefore they conclude that melanocytes become sensitive to the oncogenic effect of *GNAQ(Q209L)* only during certain temporary phases of their development. Thus, these results suggest an essential role of *GNAQ* mutations in the tumorigenesis of the melanocyte lineage.

Nevertheless, these data do not explain the *GNAQ*/*GNA11* selective mutational pressure observed in uveal melanoma compared to cutaneous melanoma or why they are considered to be oncogenic [17,19]. Uveal melanoma is a subtype of melanoma which develops from melanocytes located in the eye, and its incidence is generally much lower than that of cutaneous melanoma [51]. *GNAQ* and *GNA11* mutations were amplified by real-time PCR in the circulating DNA from the plasma of 22 patients with uveal melanoma, and Q209 mutations were detected by ultradeep sequencing in 9 of these [52]. More recently, the identification of additional somatic mutations in uveal melanoma which could not be detected by classical NGS led to a tumor classification in four molecular and clinical subgroups that will allow a stratification of uveal melanoma patient management. Moreover, “secondary” driver mutations in the GNA pathway have also been detected in uveal melanoma at low frequencies, but might account for tumor development and progression [53,54]. Of note, a retrospective study identified 18 patients with metastatic *GNAQ/11* mutant nonuveal melanoma, which showed lower tumor mutational burden and fewer ultraviolet signature mutations than cutaneous melanomas. In contrast to uveal melanoma, these tumors frequently metastasized lymphatically and concurrent mutations (*EIF1AX*, *SF3B1* and *BAP1)* were not associated with patient prognosis. The authors concluded that primary *GNAQ/11* mutant nonuveal melanoma is a subtype of melanoma that is clinically and genetically distinct from both cutaneous and uveal melanoma [55].

The selective mutational rate in uveal melanoma compared to cutaneous melanoma is not well understood. One hypothesis could be a particular oncogenic activity of these mutations in ocular melanocytes, since most cutaneous melanocytes tend to become senescent when carrying *GNA* mutations. Indeed, as discussed above, *GNAQ* or *GNA11* are mutated in nearly 83% blue naevi [17,19]. 

In uveal melanoma, hot spot somatic mutations in *GNAQ*/*GNA11* lead to amino acid substitutions in exon 5 (p.Q209L or p.Q209P) or in exon 4 (p.R183C). While the first group of mutations prevents the intrinsic GTPase activity and therefore constitutively activates the protein, the second group leads to only a partial loss of GTPase activity. 

In the first described uveal melanoma mouse model, an oncogenic *Gnaq* mutant under the control of the *Rosa26* promoter was conditionally expressed by the *cre* recombinase under the control of melanocyte specific-promoter *Mitf,* and could initiate tumors which progressed to intravasation and metastases in 100% offspring [56]. In addition, the YAP protein (Hippo pathway) could play a role in this process. Interestingly, the overexpression of mutant *Gnaq(Q209L)* led to a loss of cutaneous melanocytes in adult mice, which could explain why this mutation is not found in cutaneous melanoma.

In another mouse model, melanocyte-specific expression of *Gna11(Q209L)* led to pigmented neoplastic lesions from melanocytes of the skin and noncutaneous organs, including the eye and leptomeninges [57]. Additional loss of *Brca-1-associated protein* (*Bap1*) increased the size of cutaneous melanoma tumors. *BAP1* is a chromatin-associated deubiquitinase, and the function of which is involved in DNA double-strand breaks repair [58,59]. However, *BAP1*-inactivating mutations appear in 40% of uveal melanoma. The author also identified *RasGRP3* expression specifically in *Gnaq/Gna11*-driven melanoma and observed that *RasGRP3* is required for *Gnaq/Gna11*-driven RAS activation. 

Interestingly, other members in the GNA signaling have also been identified with mutations specifically in uveal melanoma. One example is the GPCR cysteinyl leukotriene receptor 2 (CYSLTR2), a member of the rhodopsin-like family that responds to purinergic or pyrimidinergic nucleotides (P2Ys) [60]. Recurrent mutations on codon 129 and 136 have been identified in uveal melanoma, but only the first one is oncogenic [61]. CYSLTR2 is activated by lipid mediators called leukotrienes, which can not only induce a strong cutaneous melanocyte proliferation but also induce cancer [62,63]. The phospholipase C (PLC) family represents one of the downstream targets of GNA proteins. The role of phospholipases is to hydrolyze PIP2 into the following second messengers: IP3, which controls intracellular calcium concentration and DAG, which activates PKC (protein kinase C), which in turn activates MAPK to control cell proliferation. Mutations in members of this family, PLCB4 and PLCB3, were identified in patient-derived uveal melanoma samples [61,64,65]. However, their exact function as driver oncogenes is not yet clear. Another example of the downstream target is the identification of *TRIO* (GEF) in a genome-wide RNAi screen being essential in the signal transduction from GNAQ to the nucleus, independently of PLCB. Moreover, this signal transduction activated RHO- and RAC-regulated pathways acting on JNK and p38 [66]. Although *TRIO* is found altered in 10% cutaneous melanoma, its potential role in the development of this cancer type is not yet demonstrated.

## 6. Targeting the GNA Pathway as a Therapeutic Option for Uveal Melanoma

Uveal melanoma is a very resistant cancer to classical chemotherapy or to radiotherapy. Immune checkpoint inhibitors which have greatly improved the overall survival of patients with advanced cutaneous melanoma, unfortunately showed very little efficacy in metastatic uveal melanoma [67,68,69,70]. However, combined checkpoint blockade represents the most effective treatment option available so far for metastatic uveal melanoma outside of clinical trials [71,72].

The location of the eye as an immune privilege organ may account for the loss of specific immune response [73]. In addition, the low mutational burden of uveal melanoma (and therefore the weak generation of neoantigens) might also play a role in this lack of response to immunotherapies [64,74]. Since no efficient treatment for patients with metastatic uveal melanoma is available at the moment, immunotherapy treatments in combination with targeted therapies could be an alternative worthy of clinical trials.

The combination of BRAF/MEK inhibitors is not possible due to the absence of *BRAF* mutations in uveal melanoma [75,76,77]. Clinical trials for MEK inhibitors in combination with chemotherapy or other candidate targets (PKC, AKT) have shown no significant improvement in the progression free survival [78,79]. Indeed, the observed heterogeneity of MAPK activation in uveal melanoma with *GNAQ/11* mutations could explain at least in part this phenomenon and, therefore, it does not allow this mutational status to be an efficient biomarker of MEK inhibitors’ sensitivity [80].

Although a direct link between the activation of G proteins/GPCRs and tumor development has been proven, no therapeutic strategy targeting the function of G proteins is currently available in clinics [81]. Nevertheless, in light of the data discussed above, it appears quite obvious that the inhibition of oncogenic G proteins and their downstream targets should lead to the efficient killing of tumors that developed from driver *GNA* mutations [82]. 

**Figure 2:**
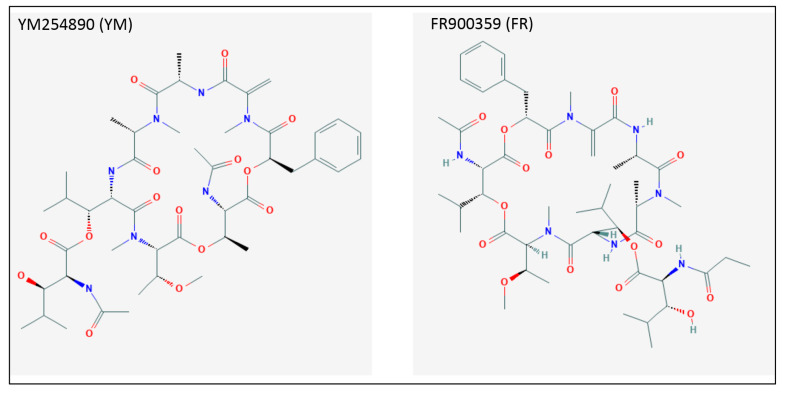
Chemical structure of GNAQ inhibitors FR900359 (FR) and YM254890 (YM).

The so-called guanine nucleotide dissociation inhibitors (GDI) are molecules that are able to maintain G proteins in their GDP-bound inactive state (Figure 1). Of these, GNAQ family-specific inhibitors FR900359 (FR) and YM254890 (YM) were described to block the downstream signaling of GNAQ variants in cancer cells with high GNAQ activity [83]. Several studies showed under FR treatment a significant reduction in the aggressive phenotype in skin melanoma cells, proliferation of which was dependent on GNAQ [22,84,85,86]. The chemical structure of FR and YM are represented in Figure 2.

In uveal melanoma-specific mutations, both identified hotspots are located in the GTPase domain and play a key role in stabilizing the transition state for GTP hydrolysis. As discussed above, these variants differ because the R183C variant is still able to be regulated by receptor stimulation, whereas Q209L/P variants are uncoupled from GPCRs [87,88].

Based on this result, one should assume a different sensitivity to GDIs depending on which variant is expressed in the tumor cells. One advantage of GDIs could be their dual role of blocking signaling downstream wildtype but also oncogenic G proteins.

The path of GDI into the clinic still needs to cross several steps. Safety issues such as the ubiquitous expression of G proteins could be overcome by local treatment instead of systemic (for example, directly into the eye for uveal melanoma). Documentation on the long term toxicity in humans is still missing. Finally, this “mutation-nonspecific” type of inhibitor will block both oncogenic and wildtype forms of the protein, which will require precise adjustment of the moelcule’s dosage.

CYSLTR2-mutated uveal melanoma usually presents an overactivation of the GNAQ pathway and an insensitivity to any ligand [89]. Specific antagonists of CYSLTR2 have been generated in 2010 [90] and CYSLTR1/2 inhibitors have been tested in clinical trials for the condition of asthma. The latter led to a significant attenuation of allergen-induced inflammation in the tested cohort [91].

It is therefore likely that these inhibitors will be tested in uveal melanoma with GPCR mutations in the future. GDIs such as FR or YM are also expected to have an impact on tumors with aberrant activation of the G proteins, whether they carry a mutation on *GNA* genes or upstream their receptor.

Mutations in *GNAQ* and *CYSTR2* represent the vast majority of uveal melanoma tumors and they activate downstream targets such as *TRIO* or *ARF6*. Inhibitors of these two proteins have been developed, and it would make sense to test them in uveal melanoma [92,93,94]

## 7. Conclusions

The observation that one of the direct causes for cancer development is the alteration of genes encoding for G proteins, leading to inactivate their GTPase function, has been known for a while. Nonetheless, trials for inhibiting these oncoproteins have brought very little success for biochemical reasons. Indeed, unlike cell membrane receptors, G proteins are intracellular and more difficult to access. In addition, unlike the success of ATP binding competition in the case of kinase inhibitors, the high affinity of GTP/GDP to the protein together with their high intracellular levels renders the chemical competition difficult. The development of specific inhibitors for GNAQ, efficacy of which was shown in vitro and in vivo, has paved the road for a rational therapeutic approach in cancers carrying alterations in this particular protein.

The field of melanoma research has made unprecedented significant advances in the last decade (targeted and immunotherapies), which resulted in a prolongation of the survival for patients with advanced cutaneous melanoma. However, in the case of uveal melanoma, these new therapeutic strategies have not yet led to major improvements due to two main reasons. First, the major risk factor for melanoma, UV radiation from the sun, does not play a big role in the development of uveal melanoma. Second, uveal melanoma transformation generates from different oncogenic drivers than cutaneous melanoma. The vast majority of tumors carry activating mutations in *GNAQ/11*, which leads to the overactivation of signaling such as ARF6/TRIO/RHO/RAC/YAP and PLCB/PKC/ERK. In addition, 40% uveal melanoma present genetic alterations in *BAP1*, which are associated with metastasis [95].

Based on the late advances in both the molecular mechanisms of uveal melanoma development and possibilities of targeting G protein signaling in this cancer type, we expect to observe an improvement in therapeutic strategies for patients with advanced uveal melanoma in the near future.

## Figures and Tables

**Table 1:** Frequency of *GNA* alterations in cancers.

Cancers	*GNAS*					
	Alteration	Mutation	Fusion	Amplification	Deep deletion	Multiple Alterations
Colorectal	11.45% of 594 cases	3.87% (23 cases)	0.17% (1 case)	7.24% (43 cases)		0.17% (1 case)
Stomach	9.55% of 400 cases	5.45% (24 cases)	3.64% (16 cases)	0.23% (1 case)	0.23% (1 case)	
Uterine	9.07% of 529 cases	7.18% (38 cases)		1.89% (10 cases)		
Lung adeno	7.77% of 566 cases	3.71% (21 cases)	3.89% (22 cases)	0.18% (1 case)		
Esophagus	7.69% of 182 cases	4.95% (9 cases)		2.75% (5 cases)		
Melanoma	7.21% of 444 cases	6.08% (27 cases)		1.13% (5 cases)		
Pancreas	7.07% of 184 cases	4.89% (9 cases)	0.54% (1 case)	1.63% (3 cases)		
Sarcoma	7.06% of 255 cases	1.57% (4 cases)		5.49% (14 cases)		
Uterine CS	7.02% of 57 cases	3.51% (2 cases)		3.51% (2 cases)		
ACC	6.59% of 91 cases	5.49% (5 cases)		1.1% (1 case)		
BICB	6.18% of 1084 cases	1.01% (11 cases)	0.37% (4 cases)	4.61% (50 cases)	0.18% (2 cases)	
Ovarian	5.14% of 584 cases	0.86% (5 cases)	0.17% (1 case)	3.94% (23 cases)		0.17% (1 case)
Cervical	4.38% of 297 cases	3.03% (9 cases)	0.34% (1 case)	1.01% (3 cases)		
Bladder	3.41% of 411 cases	2.68% (11 cases)	0.49% (2 cases)	0.24% (1 case)		
Head & Neck	2.68% of 523 cases	2.49% (13 cases)		0.19% (1 case)		
Lung squ	2.46% of 487 cases	1.85% (9 cases)	0.21% (1 case)	0.21% (1 case)	0.21% (1 case)	
Liver	2.42% of 372 cases	1.34% (5 cases)		1.08% (4 cases)		
PCPG	1.69% of 178 cases	1.12% (2 cases)		0.56% (1 case)		
Thyroid	1.2% of 500 cases	0.8% (4 cases)	0.4% (2 cases)			
Mesothelioma	1.15% of 87 cases	1.15% (1 case)				
pRCC	1.06% of 283 cases	1.06% (3 cases)				
Prostate	0.81% of 494 cases			0.61% (3 cases)	0.2% (1 case)	
Testicular germ cell	0.67% of 149 cases	0.67% (1 case)				
ccRCC	0.59% of 511 cases	0.59% (3 cases)				
LGG	0.58% of 514 cases	0.19% (1 case)		0.39% (2 cases)		
GBM	0.51% of 592 cases	0.51% (3 cases)				
AML	0.5% of 200 cases	0.5% (1 case)				
Cholangiocarcinoma						
DLBC						
Kidney Chromophobe						
Thymoma						
**Uveal melanoma**						
	***GNAQ***					
	**Alteration**	**Mutation**	**Fusion**	**Amplification**	**Deep deletion**	**Multiple Alterations**
**Uveal melanoma**	**50% of 80 cases**	**50% (40 cases)**				
Uterine	3.97% of 529 cases	2.84% (15 cases)		0.38% (2 cases)	0.76% (4 cases)	
Melanoma	3.38% of 444 cases	3.38% (15 cases)				
Stomach	2.5% of 440 cases	0.91% (4 cases)		0.23% (1 case)	1.36% (6 cases)	
Esophagus	2.2% of 182 cases	0.55% (1 case)		0.55% (1 case)	0.55% (1 case)	0.55% (1 case)
DLBC	2.08% of 48 cases				2.08% (1 case)	
Bladder	1.95% of 411 cases	0.73% (3 cases)		0.24% (1 case)	0.97% (4 cases)	
Uterine CS	1.75% of 57 cases	1.75% (1 case)				
Sarcoma	1.57% of 255 cases			1.57% (4 cases)		
Colorectal	1.52% of 594 cases	1.35% (8 cases)		0.17% (1 case)		
Lung adeno	1.41% of 566 cases	0.88% (5 cases)			0.53% (3 cases)	
Ovarian	1.37% of 584 cases			0.68% (4 cases)	0.68% (4 cases)	
ACC	1.1% of 91 cases			1.1% (1 case)		
Pancreas	1.09% of 184 cases	0.54% (1 case)			0.54% (1 case)	
GBM	1.01% of 592 cases	0.17% (1 case)		0.68% (4 cases)	0.17% (1 case)	
Cervical	1.01% of 297 cases	0.67% (2 cases)			0.34% (1 case)	
BICB	1.01% of 1084 cases	0.28% (3 cases)	0.09% (1 case)	0.18% (2 cases)	0.37% (4 cases)	0.09% (1 case)
Lung squ	0.82% of 487 cases	0.41% (2 cases)			0.41% (2 cases)	
Liver	0.81% of 372 cases	0.27% (1 case)		0.27% (1 case)	0.27% (1 case)	
Thymoma	0.81% of 123 cases				0.81% (1 case)	
Head & Neck	0.76% of 523 cases			0.38% (2 cases)	0.38% (2 cases)	
Testicular germ cell	0.67% of 149 cases	0.67% (1 case)				
PCPG	0.56% of 178 cases			0.56% (1 case)		
AML	0.5% of 200 cases				0.5% (1 case)	
pRCC	0.35% of 283 cases				0.35% (1 case)	
Thyroid	0.2% of 500 cases				0.2% (1 case)	
Prostate			0.4% (2 cases)			
LGG						
Cholangiocarcinoma						
Kidney Chromophobe						
ccRCC						
Mesothelioma						
	***GNA11***					
	**Alteration**	**Mutation**	**Fusion**	**Amplification**	**Deep deletion**	**Multiple Alterations**
**Uveal melanoma**	**46.25 % of 80 cases**	**45% (36 cases)**		**1.25% (1 case)**		
Sarcoma	5.88% of 255 cases	0.39% (1 case)	0.39% (1 case)	3.53% (9 cases)	1.57% (4 cases)	
Cervical	4.71% of 297 cases	1.01% (3 cases)		1.35% (4 cases)	2.36% (7 cases)	
Melanoma	4.05% of 444 cases	3.83% (17 cases)		0.23% (1 case		
Esophagus	3.3% of 182 cases			1.1% (2 cases)	2.2% (4 cases)	
Ovarian	2.91% of 584 cases	0.51% (3 cases)			2.4% (14 cases)	
Uterine	2.84% of 529 cases	1.89% (10 cases)			0.95% (5 cases)	
LGG	2.33% of 514 cases	0.19% (1 case)		2.14% (11 cases)		
Lung adeno	1.59% of 566 cases	0.71% (4 cases)		0.18% (1 case)	0.71% (4 cases)	
Colorectal	1.52% of 594 cases	1.01% (6 cases)			0.51% (3 cases)	
Bladder	1.46% of 411 cases	0.73% (3 cases)		0.73% (3 cases)		
BICB	1.29% of 1084 cases	0.46% (5 cases)		0.18% (2 cases)	0.65% (7 cases)	
Prostate	1.21% of 494 cases	0.2% (1 case)	0.2% (1 case)		0.81% (4 cases)	
GBM	1.18% of 592 cases	0.17% (1 case)		0.84% (5 cases)	0.17% (1 case)	
Mesothelioma	1.15% of 87 cases			1.15% (1 case)		
PCPG	1.12% of 178 cases			1.12% (2 cases)		
Pancreas	1.09% of 184 cases	0.54% (1 case)		0.54% (1 case)		
Liver	0.81% of 372 cases	0.27% (1 case)			0.54% (2 cases)	
Thymoma	0.81% of 123 cases			0.81% (1 case)		
Head & Neck	0.57% of 523 cases	0.38% (2 cases)			0.19% (1 case)	
AML	0.5% of 200 cases				0.5% (1 case)	
Lung squ	0.41% of 487 cases	0.21% (1 case)			0.21% (1 case)	
ccRCC	0.39% of 511 cases				0.39% (2 cases)	
ACC						
Cholangiocarcinoma						
DLBC						
Kidney Chromophobe						
pRCC						
Stomach						
Testicular germ cell						
Thyroid						
Uterine CS						

Data were obtained in cBioportal from the studies TCGA PANCAN2018 (cerami et al. 2012; gao et al. 2013) [12,24]. AML: Acute Myeloid Leukemia, ACC: Adrenocortical Carcinoma, LGG: Brain Lower Grade Glioma, DLBC: Diffuse Large B-Cell Lymphoma, GBM: Glioblastoma Multiforme, ccRCC: Kidney Renal Clear Cell Carcinoma, pRCC: Kidney Renal Papillary Cell Carcinoma, PCPG: Pheochromocytoma and Paraganglioma, BICB: Breast Invasive Carcinoma Breast.
